# Discrepancy between prevalence and perceived effectiveness of treatment methods in myofascial pain syndrome: Results of a cross-sectional, nationwide survey

**DOI:** 10.1186/1471-2474-11-32

**Published:** 2010-02-11

**Authors:** Johannes Fleckenstein, Daniela Zaps, Linda J Rüger, Lukas Lehmeyer, Florentina Freiberg, Philip M Lang, Dominik Irnich

**Affiliations:** 1 Multidisciplinary Pain Centre, Department of Anaesthesiology, University of Munich, Munich, Germany

## Abstract

**Background:**

Myofascial pain is a common dysfunction with a lifetime prevalence affecting up to 85% of the general population. Current guidelines for the management of myofascial pain are not available. In this study we investigated how physicians on the basis of prescription behaviour evaluate the effectiveness of treatment options in their management of myofascial pain.

**Methods:**

We conducted a cross-sectional, nationwide survey with a standardized questionnaire among 332 physicians (79.8% male, 25.6% female, 47.5 ± 9.6 years) experienced in treating patients with myofascial pain. Recruitment of physicians took place at three German meetings of pain therapists, rheumatologists and orthopaedists, respectively. Physicians estimated the prevalence of myofascial pain amongst patients in their practices, stated what treatments they used routinely and then rated the perceived treatment effectiveness on a six-point scale (with 1 being excellent). Data are expressed as mean ± standard deviation.

**Results:**

The estimated overall prevalence of active myofascial trigger points is 46.1 ± 27.4%. Frequently prescribed treatments are analgesics, mainly metamizol/paracetamol (91.6%), non-steroidal anti-inflammatory drugs/coxibs (87.0%) or weak opioids (81.8%), and physical therapies, mainly manual therapy (81.1%), TENS (72.9%) or acupuncture (60.2%). Overall effectiveness ratings for analgesics (2.9 ± 0.7) and physical therapies were moderate (2.5 ± 0.8). Effectiveness ratings of the various treatment options between specialities were widely variant. 54.3% of all physicians characterized the available treatment options as insufficient.

**Conclusions:**

Myofascial pain was estimated a prevalent condition. Despite a variety of commonly prescribed treatments, the moderate effectiveness ratings and the frequent characterizations of the available treatments as insufficient suggest an urgent need for clinical research to establish evidence-based guidelines for the treatment of myofascial pain syndrome.

## Background

Myofascial pain syndrome is a chronic muscular pain disorder in one muscle or groups of muscles accompanied by local and referred pain, decreased range of motion, weakness, and often autonomic phenomena. It is a primary cause of health-care visits, absenteeism and invalidity pensions [1].

Myofascial pain affects up to 85% of the general population [2]. Myofascial trigger points play a central role in the pathophysiology of common myofascial pain syndromes [3]. Myofascial trigger points are defined as hyperirritable spots, usually within a taut band of skeletal muscle or in the muscle fascia, which is painful on compression and can give rise to characteristic referred pain, motor dysfunction, and autonomic phenomena [2]. Moreover, abnormal spontaneous electrical activities have been described at these sites [4]. Several biochemical and proinflammatory mediators seem to be involved in these ectopic pain mechanisms [5,6] and were found to be significantly higher in active trigger points [5]. Neuroplastic remodelling has been shown in the peripheral and central nerve system [7]. However, the understanding of the neurophysiologic genesis of trigger points and their role in myofascial pain syndrome remains a present focus of research [8].

Treatment approaches in myofascial pain range from analgesics to various physical modalities. These approaches include combined techniques (e.g. spray and stretch), manual techniques, transcutaneous electrical stimulation (TENS), frequency-modulated neural stimulation, ultrasound or massage, injections, acupuncture and dry needling [9-14]. Analgesics are often used in the treatment of myofascial pain [15]. Despite the variety of treatment approaches, there is a lack of clinical evidence to guide treatment. There is moderate evidence suggesting that back schools reduce pain in an occupational setting and improve function and return-to-work status [16]. Acupuncture and dry-needling may be useful adjuncts [13,17-20]. Trigger point injections remain the treatment with the most scientific support [18,20,21]. Various injected substances have been investigated. These include local anaesthetics, botulinum toxin, sterile water and sterile saline [15,17,18,21,22]. Despite, data does neither favour injection of any substance in particular over injection of an inert substance, nor are injections (wet needling) superior to dry needling [18,20,23]. The available data do explicitly not support the use of botulinum toxin injection in trigger points for myofascial pain [21,22].

In conclusion there is not sufficient clinical evidence to incorporate evidence-based guide treatments.

We therefore evaluated the distribution of the usage and physicians rating of effectiveness for frequently prescribed treatments amongst German physicians with experience in treating patients with myofascial pain.

## Methods

### Design and setting

We conducted a cross-sectional, nationwide survey among German physicians involved in the management of patients with myofascial pain. Participants were recruited at the annual meetings of the German Society for the Study of Pain, German chapter of the IASP [Deutsche Gesellschaft zum Studium des Schmerzes], the Professional Organisation of German Rheumatologists [Berufsverband Deutscher Rheumatologen] and the Association of Southern German Orthopaedists [Vereinigung Süddeutscher Orthopäden]. Participants completed a standardised questionnaire.

A subgroup analysis was performed within three groups of all physicians. We chose pain therapists, orthopaedists and rheumatologists as they were thought to be the ones with most experience in attending and treating myofascial pain syndrome.

### Ethics

In this trial we consider the ethical, legal and regulatory norms and standards for research involving human subjects in Germany. As this study did not involve patient data and the research did not involve humans, ethical approval was not necessary http://www.wma.net/en/30publications/10policies/b3/index.html. Written consent was not sought from each participant for use of survey data, but consent of respondents was assumed if they completed and returned the questionnaire.

### Questionnaire (additional file 1)

#### Demographic and participants characteristics

In the first part we assessed the following demographic data: age, gender, field of specialisation (surgery, internal medicine, anaesthesiology, neurology and orthopaedics), subspecialisation (pain therapy, rheumatology and traumatology), employment centre (university hospital, county hospital, private clinic and pain centre), and status of specialisation (resident and consultant). We asked for the physician's average number of treated myofascial pain patients and the number of patients referred to specialised pain centres.

#### Estimated importance of myofascial pain syndrome and prevalence of myofascial trigger points

Secondly we wanted the physicians to estimate the importance of myofascial pain in the general population on a six-point scale (with 1 being a very common problem). Additionally the physicians had to estimate the prevalence of active trigger points in the population and their respective patients in percent.

#### Prescription rates and rating of treatment options

Physicians were asked to choose their routinely prescribed therapeutic options in the treatment of myofascial pain syndrome from a list of prespecified options. Afterwards they had to rate the effectiveness of the approaches chosen based on their own experience on a six-point scale (with 1 being "excellently effective" and 6 being "ineffective"). We asked for analgesics (non-steroidal anti-inflammatory drugs/coxibs, metamizol/paracetamol, weak and strong opioids, anticonvulsants, antidepressants,), physical medicine (TENS, manual therapy, ultrasound, percussion waves, acupuncture, dry needling) and injections (spinal interventions, injection of botulinum toxin or local anaesthetics). In each category, physicians had the opportunity to name additional treatment approaches (free text). Finally, we asked for the opinion regarding the sufficiency of available treatment options.

### Statistical analysis

All statistical analysis was carried out using the SPSS statistical software system (SPSS Inc., Chicago, IL; version 15.0). Data were expressed as mean ± SD. Between-group differences were examined with Kruskal-Wallis tests, using the Mann-Whitney U tests for post-hoc two-group comparisons. Two-sided p < 0.05 were considered statistically significant. Due to the exploratory nature of our analyses, no alpha adjustments for multiple testing were undertaken.

## Results

### Demographic and participants characteristics

Three hundred thirty-two physicians with a mean age of 47.5 ± 9.6 years (85 female, 25.6%, 43.6 ± 9.2 years; 235 male, 70.8%, 48.8 ± 9.4 years) responded to the questionnaire. Among all physicians, 146 (44.0%) were orthopaedists, 97 (29.2%) internists and 63 (19.0%) anaesthetists. Out of these groups, 90 (27.1%) were sub-specialised in rheumatology and 50 (15.1%) in pain therapy. Thirty-seven (11.1%) doctors worked at a university hospital, 113 (34.0%) at a county hospital, 160 (48.2%) in a private clinic, 2 (0.6%) in a pain centre and 20 (6.0%) did not specify their place of work. Forty-one (12.3%) physicians were residents and 281 (84.6%) consultants. The detailed affiliations and professional status of the respondents are shown in Table S1 (additional file 2).

Congress groups showed differences regarding age, gender and the work centre. Female rheumatologists were younger than male rheumatologists. In general, women were not only younger but also differently distributed within the groups of specialisation and subspecialisation when compared to men. There were less female surgeons than female rheumatologists or anaesthetists. Physicians working in a hospital were younger than those in a private clinic.

When asked for the number of myofascial pain patients treated, 163 (48.9%) of all physicians attended more then four patients a week, 87 (26.1%) between one and three patients a week, 39 (11.7%) between one and three patients per month, 36 (10.8%) up to ten patients a year and 6 (1.8%) physicians never saw myofascial pain patients. There were no intergroup differences regarding those distributions (p = 0.801).

When we asked how often patients were referred to special pain centres, we found the following distribution: 82 (24.6%) of physicians never did, 89 (26.7%) up to ten patients per year, 96 (28.8%) up to three patients per month, 25 (7.5%) between one and three patients a week and 26 (7.8%) referred more than four patients a week to a specialised pain centre. Pain therapists rarely refer their patients to another centre (p < 0.001). For detailed information see Table S1 (additional file 2).

### Estimated importance of myofascial pain syndrome and prevalence of myofascial trigger points

When asked for the importance of myofascial pain the physicians mean score was 2.5 ± 1.4 (n = 330). There were no significant differences among the respective groups (p = 0.803), specialities (p = 0.578), genders (p = 0.294) or other demographic data.

Physicians estimated the prevalence of active trigger points to 46.1 ± 27.4% (n = 329) in the overall population and 52.8% ± 26.9 (n = 330) in their own patients. Subjects from the pain congress rated these frequencies higher than other physicians; 55.4 ± 22.2% in the overall population (p < 0.001) and 63.4 ± 21.7% in their own patients (p = 0.001). For detailed information see Table S2 (additional file 3).

### Prescription Rates of treatment options

#### Analgesics

**Figure 1:**
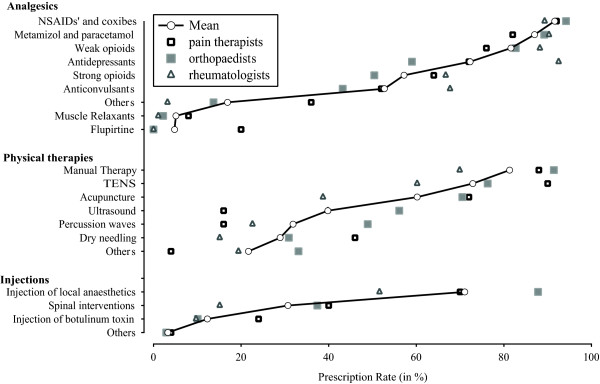
**Prescription Rate**. demonstrates the physician estimated prescription rate of different therapeutic options in the treatment of myofascial pain. Circles indicate the overall average; the prescription rates of three subgroups (Pain therapists, rheumatologists and orthopaedists) are shown by triangles or squares (please refer to legend). Data are expressed in percent (%). TENS: transcutaneous electrical stimulation.

Pharmacological approaches were the most common treatment (1525 choices, i.e. a mean of 4.5 analgesics evaluated per physician). Non steroidal anti-inflammatory drugs or coxibs were the main analgesics (n = 304, 91.6%), followed by metamizol and paracetamol (n = 289, 87.0%), weak opioids (n = 271, 81.6%), antidepressants (n = 240, 72.3%), strong opioids (n = 190, 57.2%) or anticonvulsants (n = 175, 52.7%). Other drugs were rarely used (n = 56, 16.9%), physicians named especially muscle relaxants (n = 17, 5.1%) and flupirtine (n = 16, 4.8%). Detailed intra- and inter-group values are shown in Figure 1 and Table S3 (additional file 4).

#### Physical therapy

Physical therapies were prescribed one-third less often than analgesics (1118 choices, i.e. a mean of 3.4 physical therapies evaluated per physician). Manual therapy was prescribed most often, i.e. by 270 (81.1%) of all physicians, followed by TENS (n = 242, 72.9%) and acupuncture (n = 200; 60.2%). Ultrasound (n = 132, 39.8%), percussion waves (n = 106, 31.9%) or dry needling (n = 96, 28.9%) were prescribed less often. Additional treatments were chosen by 72 (21.7%) of all physicians. These treatments were rather specified e.g. chiropractics (n = 8), cryotherapy (n = 3) and osteopathy (n = 3) as other physical therapy. Detailed intra- and inter-group values are shown in Figure 1 and Table S3 (additional file 4).

#### Injections

The use of injection techniques is less important in the overall therapeutic concept (390 choices, i.e. a mean of 1.2 injections evaluated per physician). Injection of local anaesthetics was mainly used (n = 236; 71.1%). Spinal interventions (e.g. spinal cord stimulation, epidural injection) was used by 102 (30.7%) whereas injection of botulinum toxin was used by 41 (12.3%) of physicians. Additional techniques (n = 11, 3.3%) were not specified. Detailed intra- and inter-group values are shown in Figure 1 and Table S3 (additional file 4).

### Rating of treatment approaches

A 6-point scale (with 1 being excellent effective and 6 being worst) allowed physicians to rate the effectiveness of the used treatment approaches. 54.3% of all physicians surveyed stated current symptom-based treatment options being insufficient. This opinion was more pronounced in the rheumatologists group (77.3%) than anaesthetists (49.2%) or orthopaedists (45.8%).

#### Analgesics

**Figure 2:**
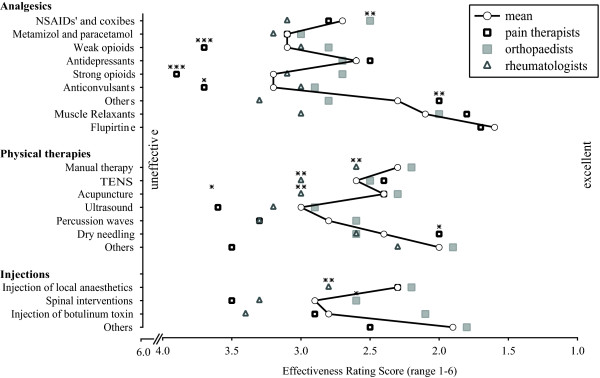
**Ratings of Treatment Options**. demonstrates the physician estimated efficacy of different therapeutic options in the treatment of myofascial pain on a 6-fold scale (with 1 being "excellently effective" and 6 being "ineffective"). Ratings towards the lower ranks on the value axis indicate a higher estimated effectiveness. Data are expressed as mean ± SD. (*), (**) and (***) express the different levels of significance p < 0.05, 0.01 and 0.001. Between-group differences were examined with Kruskal-Wallis tests, using the Mann-Whitney U tests for post-hoc two-group comparisons. Stars are placed upon the confirmed group, respectively. TENS: transcutaneous electrical stimulation.

Muscle relaxants (2.1 ± 0.5) and flupirtine (1.6 ± 0.7) are estimated as most effective analgesics. They form part of additional assignable drugs (overall 2.3 ± 1.1 points). They were followed by antidepressants (2.6 ± 1.0), non-steroidal anti-inflammatory drugs or coxibs (2.7 ± 1.0), metamizol or paracetamol (3.1 ± 1.0), weak opioids (3.1 ± 1.1), strong opioids (3.2 ± 1.5) and anticonvulsants (3.2 ± 1.2). Detailed intra- and intergroup data are shown in Figure 2 and Table S4 (additional file 5).

There were significant intragroup differences regarding the rating of treatment options for non-steroidal anti-inflammatory drugs or coxibs (p = 0.002), weak and strong opioids (p < 0.001), anticonvulsants (p = 0.024) and additional pharmacologic approaches (p = 0.006). Non-steroidal anti-inflammatory drugs or coxibs were rated better by orthopaedists when compared to pain therapists (p = 0.018) or to rheumatologists (p = 0.001); weak and strong opioids were rated less effective by pain therapists than orthopaedists (p < 0.001) or rheumatologists (p < 0.01); pain therapists rated anticonvulsants worse than orthopaedists (p = 0.008); Other pharmacologic treatments scored better in the pain group then with orthopaedists (p = 0.007) or rheumatologists (p = 0.018).

#### Physical therapies

Manual therapy was rated with an average of 2.3 ± 0.9. Dry needling (2.4 ± 1.1) and acupuncture (2.4 ± 1.0) were estimated with a similar effectiveness. TENS (2.6 ± 0.9) scored better than percussion waves (2.8 ± 1.2). Ultrasound techniques were estimated being the less effective therapeutic option (3.0 ± 1.2). Additional treatments (e.g. chiropractics, osteopathy) were rated 2.0 ± 0.9. Detailed intra- and intergroup data are shown in Figure 2 and Table S4 (additional file 5).

There were intragroup differences regarding the rating of TENS (p = 0.001), manual therapy (p = 0.002) and acupuncture (p = 0.006). TENS got a better ranking by orthopaedists (p = 0.001) and pain therapists (p < 0.001) when compared to rheumatologists. The same held for manual therapy (p = 0.001, p = 0.1) and acupuncture (p = 0.008, p = 0.002). Ultrasound was rated better by orthopaedists than pain therapists (p = 0.033); Dry needling was rated better by pain therapists than orthopaedists (p = 0.039) or rheumatologists (p = 0.044).

#### Injections

Injection of local anaesthetics was rated with an average of 2.3 ± 1.0 and injection of botulinum toxin scored 2.8 ± 1.4. Spinal interventions scored worst (2.9 ± 1.8). Additional but unspecified injections scored 1.9 ± 0.5. Detailed intra- and intergroup data are shown in Figure 2 and Table S4 (additional file 5).

There were intragroup differences regarding the rating of injection of local anaesthetics (p = 0.008). This concerned the rheumatologists group when compared to pain therapists (p = 0.032) or orthopaedists (p = 0.002). Injections were rated differently by orthopaedists and rheumatologists (p = 0.021).

#### Correlations between participant characteristics and treatment approaches

A gender effect could be demonstrated for the use of spinal interventions (p = 0.049), which is rarely used by women, while they prefer manual therapy (p = 0.019).

Physicians younger than 35 years rated the use of weak (p = 0.002) and strong opioids (p = 0.009) less effective than those older than 65 years.

Working place-related effects appeared different: physicians employed at district hospitals (n = 113) estimated non-steroidal anti-inflammatory drugs or coxibs more effective than physicians working in a private practice (n = 160, p = 0.018).

Comparing the ratings of residents and consultants, it became evident that residents evaluated weak (p = 0.004) and strong opioids (p = 0.001), antidepressants (p = 0.049) and dry needling (p = 0.042) less effective.

## Discussion

To our knowledge, the present study is the first physicians survey that estimates the prevalence and clinical importance of myofascial pain and the utilization rate and physician rated effectiveness of the most common treatment options. Our data suggests that German physicians that treat various pain issues considered myofascial pain a highly prevalent condition. Though, prescription rate and estimated effectiveness of prescribed treatments showed significant discrepancies: the most frequently prescribed treatments were not rated the most effective (Figures 1, 2). In addition, our data revealed significant discrepancies regarding the treatment of myofascial pain in different fields of specialisation.

### Implications for treatment options

This current survey demonstrates that physicians estimated the effectiveness of frequently prescribed analgesics as unsatisfactory. For example commonly used analgesics such as NSAID and coxibs, metamizol and paracetamol and weak opioids were estimated moderately in their effectiveness. This lack of concordance between frequently prescribed treatments and perceived effectiveness reflects the lack of scientific evidence and available treatment guidelines. Consensus is also requisite among researchers to define and describe myofascial pain using standard terminology and validated examination techniques [15]. In part, our observations could relate to the confusion and controversy among physicians regarding the treatment of myofascial pain disorders. At least, according to our survey, physicians seem to be aware of the ambiguity of their treatment approaches effectiveness. However Wheeler reports that most experts recommend medication as an adjunctive treatment to injection and exercise therapy in acute and chronic musculoskeletal pain.

Similar observations have been made by Tsang and her colleagues in the treatment of chronic vascular disease. A knowledge gap caused by lack of awareness of the evidence or familiarity with current treatments leads to a so called evidence-to-practice care gap [24].

We observed that the perceived effectiveness of some treatments that require specialized education and additional experience, including acupuncture, TENS, dry needling or injections varied among specialist. In general, these techniques were rated to be controversial; especially by physicians not familiar with these practical treatments. For example, ratings for injection techniques (e.g. spinal interventions) differed in between groups, what could be explained by a particular enhanced use of invasive methods in the respective clinical fields. The choice of treatment might depend on the professional career of the physicians. For example orthopaedists, by specialisation familiar with surgical techniques, were estimating injections better. In contrast, rheumatologists, familiar with pharmacologic treatments, estimated pharmacologic approaches better. There were also differences regarding the rating of group related treatments such as ultrasound and percussion waves that could be due to different educational access. Taken together these data suggest that the estimated efficacy of treatment options for myofascial pain syndrome is mostly influenced by the speciality-related training and education of the physicians.

In addition our data show that other physician-related parameters may influence the choice of treatment. We analysed the impact of working place or status of specialisation. While physicians in the pain congress group mainly worked in hospitals, those members that had chosen a private practice as their working place rated treatments differently, which might reflect the differences in work experience in these groups. It might also influence the choice and rating of treatments. We could also demonstrate that use and evaluation of treatments are influenced by the status of specialisation. It might also be possible that previously learned strategies are kept out of mind. It has been shown that physicians reconsider the use of evidence-based treatments by simple patient-specific prompting [24].

Access to education in the treatment of myofascial pain seems to be an important fact whilst improving diagnosis and treatment of myofascial pain. Bishop et al. could recently demonstrate diversity of the attitudes of general practitioners and physiotherapists in the United Kingdom (UK) towards low back pain patients. Against the line of clinical guidelines a quarter of respondents believed in avoidance of physical activity or the need to be off work as part of treatment regimen [25]. This suggests that the lack of availability of recommendations or expert opinions to health professionals is a widespread phenomenon. As a corollary, those wishing to accelerate the adoption of new evidence may need to undertake more active promotion [26], in order to obtain generally accepted guidelines [27].

### Implications for clinical practice and for future research

It remains interesting why physicians estimated seldom used pharmacologic remedies as very effective, e.g. muscle relaxants, chiropractics or flupirtine. Flupirtine for example appeared as a scientifically promising but not yet prevalent treatment option [28]. Large clinical trials for the use of flupirtine in the treatment of myofascial pain are lacking. Yet, prescription patterns and perceived effectiveness of these rarely used treatments in the view of treating physicians have still to be evaluated. To conclude, a facilitated access to the latest outcome in pain research might be desirable to get an overview of potential treatment options.

The majority of physicians, even whilst prescribing, characterized the available symptomatic treatment options as insufficient. This might also reflect the challenge to understand the sophisticated pathogenic pathways that may lead to myofascial pain syndromes. The complexity of pathogenesis might also be expressed regarding the multitude of different available treatment approaches. This survey did e.g. not approach the psychosocial aspects of treatments event though it is supposed that the occurrence of depression has been related to pain syndromes of the joints and musculoskeletal system [29]. It is commonly understood that chronic myofascial pain may be a consequence of a complex stress response that extends beyond the nervous system and contributes to the experience of pain [30].

In addition, physicians estimated the prevalence of active trigger points. Almost every second person is considered having active trigger points, thus supporting physicians opinion considering myofascial pain a highly prevalent condition. Myofascial trigger points have been described as a 'common cause of pain in clinical practice' and an 'extremely common, yet commonly overlooked' source of musculoskeletal disorders [31]. The evidence of Simon and Travells statement, as well as our results based on physicians opinions and experience, is limited as there is no available diagnostic gold standard for mTrPs based on a laboratory test or imaging technique [3]. Diagnosis is confirmed by clinical history and physical examination, free from systemic inflammatory, autoimmune or other locomotor system disease.

Clinically, an active (symptom-producing) central myofascial trigger point can be defined as a hyperirritable nodule of spot tenderness in a palpable taut band of skeletal muscle. The spot is a site of exquisite tenderness to palpation from which a local twitch response can be elicited when appropriately stimulated, that refers pain to a distance, and that can cause distant motor and autonomic effects.

These clinically findings have not been proven to be reliable, as stated in a recent review by Lucas et al. [32].

However there is no validated list of diagnostic criteria for mTrPs. Two pilot studies with a small number of subjects evaluating various diagnostic tests [33,34] reported good overall interrater reliability. They reported examining for a taut band, spot tenderness, a palpable nodule, elicited referred pain, and the local twitch response [3]. Licht et al. recently demonstrated that some 'key' diagnostic criteria acc. to Simons and Travell [31] of myofascial trigger points could reliably be found by two different examiners in a smaller sample group [35]. Harden et al. presented 2000 a list of additional 31 signs and symptoms that are related to myofascial disorders [36].

Our data demonstrate that active myofascial trigger points are considered prevalent and related to myofascial pain by the physicians. Hence, the lack of concordance in this field is limiting the conclusion how far this observation may contribute to the thesis that trigger point diagnosis is reliable.

### Methodological considerations

It is commonly understood that chronic myofascial pain may be a consequence of a complex stress response that extends beyond the nervous system and contributes to the experience of pain.

As we chose personal contact instead of postal distribution almost everyone asked responded to the questionnaire (response-rate > 95%).

In any attempt to examine the clinical practice of myofascial pain syndrome management for an entire population of physicians in a country, a questionnaire study is the most feasible method. However, we do not know to what extent the physicians' responses represent their actual practice, or whether is their perception of professional behaviour. It remains an open question whether the physicians' skills and abilities to treat myofascial pain syndrome are reflected in the answers and ratings to the questions. It is our belief that the questionnaire addresses central elements in myofascial pain management. The only reliable way to measure prescribing practices is to analyse the actual process in detail. But even then, this approach would not provide an understanding of the perceived effectiveness of the prescribed treatments.

Our questionnaire regarded the myofascial pain syndrome as a single entity. One reason was the current lack of strict classification criteria for distinct clinical entities. The participants of this study might handle different aspects of the myofascial pain syndrome and therefore choose different treatments. We took the approach on introducing questions on myofascial trigger points at the beginning of the questionnaire to help ensure that the physicians responses related to the treatment of myofascial pain syndrome rather than other soft tissue pain conditions such as fibromyalgia.

The questionnaire has not undergone formal validation. The objective of this study was to analyse and provide an overview of the diversified customs of German physicians in the treatment of myofascial pain syndrome. Given the purpose of the questionnaire, validation is not necessary.

## Conclusions

The results of this survey demonstrate that there is no agreement amongst German specialist in the utilization and perceived effectiveness of the various common treatment options for myofascial pain. In view the low rating of effectiveness, physicians seem to be aware of this drawback. We believe that guidelines for the treatment of myofascial pain syndrome are necessary. Neither standard diagnostic procedures to identify myofascial pain nor discriminating variables to distinguish the different entities of myofascial pain syndrome are available. Therefore we conclude that multiple diagnostic approaches may lead to therapeutic confusion.

All things considered, beside education in the management of myofascial pain syndrome and enhancing manual skills, clinical investigation is necessary to develop standard guidelines in the diagnosis and treatment of myofascial pain syndrome.

## Competing interests

The authors declare that they have no competing interests.

## Authors' contributions

JF conceived the study, performed the statistical analysis and drafted the manuscript. DZ, LL and FF recruited and assessed participants. LJR and PML participated in the design of the study and helped drafting the manuscript. DI participated in the design and coordination of the study and supervised drafting the manuscript. All authors read, and approved of, the final manuscript.

## Pre-publication history

The pre-publication history for this paper can be accessed here:

http://www.biomedcentral.com/1471-2474/11/32/prepub

## Supplementary Material

Additional file 1 **Questionnaire on myofascial pain**. Please find detailed description of the questionnaire within the manuscript. Click here for file

Additional file 2 **Table S1 - Demographic and participants characteristics (mean ± SD or in %)**. Table S1 provides the demographic data of physicians dealing with myofascial pain: age, gender, field of specialisation, subspecialisation, employment centre and status of specialisation. We asked for the physician's average number of treated myofascial pain patients (Treatment ratio) and the number of patients referred to specialised pain centres (Referral Ratio). Data are expressed as mean ± SD or as total count (n) and in percent (%). Click here for file

Additional file 3 **Table S2 - Estimated prevalence (mean ± SD)**. Table S2 provides the physician estimated importance of myofascial pain in the general population on a six-point scale (with 1 being a "very common problem"). Additionally the physician estimated prevalence of active trigger points in the general population and their respective patients in percent (%) is given. Data are expressed as mean ± SD. Click here for file

Additional file 4 **Table S3 - Prescription Rate of Treatment Options (in %)**. Table S3 indicates the physician estimated prescription rate of different therapeutic options in the treatment of myofascial pain. Data are expressed in percent (%). TENS: transcutaneous electrical stimulation. Click here for file

Additional file 5 **Table S4 - Ratings (1-6) of treatment options (mean ± SD)**. Table S4 indicates the physician estimated efficacy of different therapeutic options in the treatment of myofascial pain on a 6-fold scale (with 1 being "excellently effective" and 6 being "ineffective"). Data are expressed as mean ± SD. TENS: transcutaneous electrical stimulation. Click here for file

## References

[B1] Skovron ML Epidemiology of low back pain Baillieres Clin Rheumatol 1992 6 559 573 10.1016/S0950-3579(05)80127-X 1477891

[B2] Simons DG Clinical and Etiological Update of Myofascial Pain from Trigger Points Journal of Musculoskeletal Pain 1996 4 93 122 10.1300/J094v04n01_07

[B3] Simons DG Review of enigmatic MTrPs as a common cause of enigmatic musculoskeletal pain and dysfunction J Electromyogr Kinesiol 2004 14 95 107 10.1016/j.jelekin.2003.09.018 14759755

[B4] Hubbard DR Berkoff GM Myofascial trigger points show spontaneous needle EMG activity Spine 1993 18 1803 1807 10.1097/00007632-199310000-00015 8235865

[B5] Shah JP Phillips TM Danoff JV Gerber LH An in vivo microanalytical technique for measuring the local biochemical milieu of human skeletal muscle J Appl Physiol 2005 99 1977 1984 10.1152/japplphysiol.00419.2005 16037403

[B6] Mense S Hoheisel U [A lack of NO in the spinal cord as a possible factor for the occurrence of spontaneous pain] Schmerz 2001 15 19 25 10.1007/s004820170044 11810325

[B7] Mense S Nociception from skeletal muscle in relation to clinical muscle pain Pain 1993 54 241 289 10.1016/0304-3959(93)90027-M 8233542

[B8] Mense S Simons DG Russell IJ Muscle pain: its Nature, Diagnosis, and treatment 2001 Philadelphia: Lippincott, Williams & Wilkins

[B9] Bron C Wensing M Franssen JL Oostendorp RA Treatment of myofascial trigger points in common shoulder disorders by physical therapy: a randomized controlled trial [ISRCTN75722066] BMC Musculoskelet Disord 2007 8 107 10.1186/1471-2474-8-107 17983467 PMC2246010

[B10] Irnich D Trigger Point Manual [Leitfaden Triggerpunkte] 2008 Munich, Jena: Elsevier, Urban&Fischer

[B11] Lavelle ED Lavelle W Smith HS Myofascial trigger points Anesthesiol Clin 2007 25 841 851 vii-iii 10.1016/j.anclin.2007.07.003 18054148

[B12] Srbely JZ Ultrasound in the management of osteoarthritis: part I: a review of the current literature JCCA J Can Chiropr Assoc 2008 52 30 37 PMC2258240 18327300

[B13] Irnich D Behrens N Molzen H Konig A Gleditsch J Krauss M Natalis M Senn E Beyer A Schops P Randomised trial of acupuncture compared with conventional massage and "sham" laser acupuncture for treatment of chronic neck pain Bmj 2001 322 1574 1578 10.1136/bmj.322.7302.1574 11431299 PMC33515

[B14] Castien RF Windt DA van der Dekker J Mutsaers B Grooten A Effectiveness of manual therapy compared to usual care by the general practitioner for chronic tension-type headache: design of a randomised clinical trial BMC Musculoskelet Disord 2009 10 21 10.1186/1471-2474-10-21 19216763 PMC2662792

[B15] Wheeler AH Myofascial pain disorders: theory to therapy Drugs 2004 64 45 62 10.2165/00003495-200464010-00004 14723558

[B16] Heymans MW van Tulder MW Esmail R Bombardier C Koes BW Back schools for nonspecific low back pain: a systematic review within the framework of the Cochrane Collaboration Back Review Group Spine 2005 30 2153 2163 10.1097/01.brs.0000182227.33627.15 16205340

[B17] Ga H Choi JH Park CH Yoon HJ Acupuncture needling versus lidocaine injection of trigger points in myofascial pain syndrome in elderly patients--a randomised trial Acupunct Med 2007 25 130 136 10.1136/aim.25.4.130 18160923

[B18] Cummings M Baldry P Regional myofascial pain: diagnosis and management Best Pract Res Clin Rheumatol 2007 21 367 387 10.1016/j.berh.2006.12.006 17512488

[B19] Furlan AD van Tulder MW Cherkin DC Tsukayama H Lao L Koes BW Berman BM Acupuncture and dry-needling for low back pain Cochrane Database Syst Rev 2005 CD001351 15674876 10.1002/14651858.CD001351.pub2 PMC12145953

[B20] Tough EA White AR Cummings TM Richards SH Campbell JL Acupuncture and dry needling in the management of myofascial trigger point pain: a systematic review and meta-analysis of randomised controlled trials Eur J Pain 2009 13 3 10 10.1016/j.ejpain.2008.02.006 18395479

[B21] Peloso P Gross A Haines T Trinh K Goldsmith CH Burnie S Medicinal and injection therapies for mechanical neck disorders Cochrane Database Syst Rev 2007 CD000319 17636629 10.1002/14651858.CD000319.pub4

[B22] Ho KY Tan KH Botulinum toxin A for myofascial trigger point injection: a qualitative systematic review Eur J Pain 2007 11 519 527 10.1016/j.ejpain.2006.09.002 17071119

[B23] Cummings TM White AR Needling therapies in the management of myofascial trigger point pain: a systematic review Arch Phys Med Rehabil 2001 82 986 992 10.1053/apmr.2001.24023 11441390

[B24] Tsang JL Mendelsohn A Tan MK Hackam DG Leiter LA Fitchett D Lin PJ Grima E Langer A Goodman SG Discordance between physicians' estimation of patient cardiovascular risk and use of evidence-based medical therapy Am J Cardiol 2008 102 1142 1145 10.1016/j.amjcard.2008.06.037 18940280

[B25] Bishop A Foster NE Thomas E Hay EM How does the self-reported clinical management of patients with low back pain relate to the attitudes and beliefs of health care practitioners? A survey of UK general practitioners and physiotherapists Pain 2008 135 187 195 10.1016/j.pain.2007.11.010 18206309 PMC2258319

[B26] Majumdar SR McAlister FA Soumerai SB Synergy between publication and promotion: comparing adoption of new evidence in Canada and the United States Am J Med 2003 115 467 472 10.1016/S0002-9343(03)00422-4 14567371

[B27] IOM Clinical Practice Guidelines: Directions for a New Program Book Clinical Practice Guidelines: Directions for a New Program (Editor ed.êds.) 1990 City: Institute of Medicine, National Academy Press

[B28] Miceli F Soldovieri MV Martire M Taglialatela M Molecular pharmacology and therapeutic potential of neuronal Kv7-modulating drugs Curr Opin Pharmacol 2008 8 65 74 10.1016/j.coph.2007.10.003 18061539

[B29] Magni G Caldieron C Rigatti-Luchini S Merskey H Chronic musculoskeletal pain and depressive symptoms in the general population. An analysis of the 1st National Health and Nutrition Examination Survey data Pain 1990 43 299 307 10.1016/0304-3959(90)90027-B 2293141

[B30] Chapman CR Tuckett RP Song CW Pain and stress in a systems perspective: reciprocal neural, endocrine, and immune interactions J Pain 2008 9 122 145 10.1016/j.jpain.2007.09.006 18088561 PMC2278005

[B31] Simons DG Understanding effective treatments of myofascial trigger points J Bodywork Movement Therap 2002 6 81 88 10.1054/jbmt.2002.0271

[B32] Lucas N Macaskill P Irwig L Moran R Bogduk N Reliability of physical examination for diagnosis of myofascial trigger points: a systematic review of the literature Clin J Pain 2009 25 80 89 10.1097/AJP.0b013e31817e13b6 19158550

[B33] Gerwin RD Shannon S Hong CZ Hubbard D Gevirtz R Interrater reliability in myofascial trigger point examination Pain 1997 69 65 73 10.1016/S0304-3959(96)03248-4 9060014

[B34] Sciotti VM Mittak VL DiMarco L Ford LM Plezbert J Santipadri E Wigglesworth J Ball K Clinical precision of myofascial trigger point location in the trapezius muscle Pain 2001 93 259 266 10.1016/S0304-3959(01)00325-6 11514085

[B35] Licht G Müller-Ehrenberg H Mathis J Berg G Greitemann G Untersuchung myofaszialer Triggerpunkte ist zuverlässig [Examination of myofascial trigger points is reliable] Manuelle Medizin 2007 45 402 408 10.1007/s00337-007-0559-0

[B36] Harden RN Bruehl SP Gass S Niemiec C Barbick B Signs and symptoms of the myofascial pain syndrome: a national survey of pain management providers Clin J Pain 2000 16 64 72 10.1097/00002508-200003000-00010 10741820

