# Supplementary material for: Discrepancy between prevalence and perceived effectiveness of treatment methods in myofascial pain syndrome: Results of a cross-sectional, nationwide survey
Source: BMC Musculoskelet Disord. 2010 Feb 11;11:32. doi: 10.1186/1471-2474-11-32 (PMC2836281; doi:10.1186/1471-2474-11-32)
Supplement: Additional file 1 — Questionnaire on myofascial pain. Please find detailed description of the questionnaire within the manuscript. [file 1471-2474-11-32-S1.DOC]

**Fragebogen zu myofaszialen Schmerzen**

**[Questionnaire on myofascial pain]**

| **Fachrichtung [speciality]**  □ Chirurgie [surgery]  □ Traumatologie [trauma]  □ Innere Medizin [internal medicine]  □ Rheumatologie [rheumatology]  □ Anästhesiologie [anaesthesiology]  □ Neurologie [neurology]  □ Orthopädie [orthopaedics]  □ Sonstige________ [other]  □ Schmerztherapie [pain therapy] | **Tätigkeit in [employment centre]**  □ Universitätsklinik [university]  □ Krankenhaus [district hospital]  □ niedergelassen [private practice]  □ schmerztherapeutische  Einrichtung [pain centre]  □ Sonstige_____________ [other]  **Alter [age]** ____ Jahre [years] | **Ausbildungsstand [status]**  □ Arzt [resident]  □ Facharzt [consultant]  □ Berufserfahrung____Jahre  [work experience____years]  **Geschlecht [gender]**  □ m [male] □ w [female] |
| --- | --- | --- |

**1. Myofasziale Schmerzen stellen für Sie ein häufiges Schmerzproblem bei Ihren Patienten dar?**

[Myofascial pain is a prevalent condition within your patients ?]

(Bitte anhand von Schulnoten 1 – 6 beurteilen) ____________  (1 = sehr häufig, 6 = nie)

[Please specify using a six-point scale, 1 = very common, 6 = very rare]

**2. Bitte schätzen sie die Häufigkeit aktiver myofaszialer Triggerpunkte in der Bevölkerung.**

[Please estimate the prevalence of active trigger points in the population]

(Bitte anhand einer NRS von 0-100 % beurteilen) ____________  (0 = keine, 100 = gesamte Bevölkerung)

[Please specify using a 100-percent numeric rating scale, 0 = nobody, 100 = whole population]

**3. Bitte schätzen sie die Häufigkeit aktiver myofaszialer Triggerpunkte bei Ihren Patienten.**

[Please estimate the prevalence of active trigger points in your patients]

(Bitte anhand einer NRS von 0-100 beurteilen) ____________  (0 = keine, 100 = alle meine Patienten)

[Please specify using a 100-percent numeric rating scale, 0 = nobody, 100 = all patients]

**4. Wie häufig behandeln Sie Patienten mit myofaszialen Schmerzen?**

[How often do you treat patients with myofascial pain?]

□ oft (≥ 4-5 Pat./Woche)   [often; ≥ 4-5 pat per week]

□ regelmäßig (1-3 Pat/ Woche)   [regularly; 1-3 pat per week]

□ gelegentlich (1-3 Pat./Monat)   [casually; 1-3 pat per month]

□ selten (1-10 Patienten/Jahr)   [rarely; 1-10 pat per year]

□ nie [never]

**5. Wie häufig werden von Ihnen Patienten mit myofaszialen Schmerzen an eine schmerztherapeutische Einrichtung überwiesen?**

[How often do you refer patients with myofascial pain to specialised centres?]

□ oft (≥ 4-5 Pat./Woche)   [often; ≥ 4-5 pat per week]

□ regelmäßig (1-3 Pat/ Woche)   [regularly; 1-3 pat per week]

□ gelegentlich (1-3 Pat./Monat)   [casually; 1-3 pat per month]

□ selten (1-10 Patienten/Jahr)   [rarely; 1-10 pat per year]

□ nie [never]

**6. Bitte benennen und beurteilen Sie NUR die von Ihnen verwendeten Therapieoptionen bei myofaszialen Schmerzen.**

(Bitte verwendete Therapieoption(en) im Kästchen ankreuzen - Beurteilung jeweils rechts anhand von Schulnoten 1 – 6 beurteilen: 1= hochwirksam; 6 = völlig unbefriedigend)

[Please choose your routinely prescribed therapeutic options in the treatment of myofascial pain syndrome (tick the box) and rate the effectiveness of the approaches chosen based on your own experience on a six-point scale (with 1 being “excellently effective” and 6 being “ineffective”)]

| **Symptomatische Schmerztherapie**  **[symptomatic pain therapy** | | | | | **1** | | **2** | | | **3** | **4** | **5** | | **6** | | |
| --- | --- | --- | --- | --- | --- | --- | --- | --- | --- | --- | --- | --- | --- | --- | --- | --- |
| *Medikamentös*: | NSAR, Coxibe |  | **🞏** |  | |  | |  |  | |  | |  | |  |  |
| [Analgesics] | Metamizol, Paracetamol |  | **🞏** |  | |  | |  |  | |  | |  | |  |  |
|  | schwache Opiode (z.B. Tramadol)  [weak opioids] |  | **🞏** |  | |  | |  |  | |  | |  | |  |  |
|  | starke Opiode (z.B. Morphin)  [strong opioids] |  | **🞏** |  | |  | |  |  | |  | |  | |  |  |
|  | Antikonvulsiva (z.B. Gabapentin)  [anticonvulsants] |  | **🞏** |  | |  | |  |  | |  | |  | |  |  |
|  | Antidepressiva (z.B. Amitriptyllin)  [antidepressants] |  | **🞏** |  | |  | |  |  | |  | |  | |  |  |
|  | Sonstige (Bitte benennen):  [other; please specify] |  | **🞏** |  | |  | |  |  | |  | |  | |  |  |
| *Injektionen:* [injections] | Rückenmarksnah (Bitte benennen) [spinal interventions; please specify] |  | **🞏** |  | |  | |  |  | |  | |  | |  |  |
|  | Injektion von Botulinustoxin  [injection of botulinum toxin] |  | **🞏** |  | |  | |  |  | |  | |  | |  |  |
|  | Injektion von Lokalanästhetikum  [injection of local anaesthetics] |  | **🞏** |  | |  | |  |  | |  | |  | |  |  |
|  | Sonstige (Bitte benennen):  [other; please specify] |  | **🞏** |  | |  | |  |  | |  | |  | |  |  |
| *Physikalische Verfahren*:  [physical medicine] | TENS (Transkut.Elektr.Nervenstim) |  | **🞏** |  | |  | |  |  | |  | |  | |  |  |
|  | Manuelle Therapie [manual therapy] |  | **🞏** |  | |  | |  |  | |  | |  | |  |  |
|  | Ultraschall [ultrasound] |  | **🞏** |  | |  | |  |  | |  | |  | |  |  |
|  | Stoßwellen [percussion waves] |  | **🞏** |  | |  | |  |  | |  | |  | |  |  |
|  | Akupunktur [acupuncture] |  | **🞏** |  | |  | |  |  | |  | |  | |  |  |
|  | Dry needling |  | **🞏** |  | |  | |  |  | |  | |  | |  |  |
|  | Sonstige (Bitte benennen):  [other; please specify] |  | **🞏** |  | |  | |  |  | |  | |  | |  |  |

**7. Reichen die vorhandenen symptomatischen Therapieoptionen Ihrer Meinung nach aus?**

[In your opinion: are the available treatment options sufficient?]

□ ja [yes]

□ nein [no]

□ keine Meinung [no opinion]

**Vielen Dank für Ihre Mitarbeit!**
