# Supplementary material for: Discrepancy between prevalence and perceived effectiveness of treatment methods in myofascial pain syndrome: Results of a cross-sectional, nationwide survey
Source: BMC Musculoskelet Disord. 2010 Feb 11;11:32. doi: 10.1186/1471-2474-11-32 (PMC2836281; doi:10.1186/1471-2474-11-32)
Supplement: Additional file 2 — Table S1 - Demographic and participants characteristics (mean ± SD or in %). Table S1 provides the demographic data of physicians dealing with myofascial pain: age, gender, field of specialisation, subspecialisation, employment centre and status of specialisation. We asked for the physician's average number of treated myofascial pain patients (Treatment ratio) and the number of patients referred to specialised pain centres (Referral Ratio). Data are expressed as mean ± SD or as total count (n) and in percent (%). [file 1471-2474-11-32-S2.DOC]

## Table S1 - Demographic and participants characteristics (mean ± SD or in %)

Subgroups .

All Female Male Pain therapists Rheumatologists Orthopaedists

n = 332 n = 85 n =235 n = 50 n = 90 n = 139

**Gender**

Female 85 (25.6%) 21 (42.0%) 19 (13.7%) 29 (32.3%)

Male 235 (70.8%) 25 (50.0%) 120 (86.3%) 64 (71.1%)

**Mean age** (years) 47.5 ± 9.6 43.6 ± 9.2 48.8 ± 9.4 43.1 ± 6.4 48.5 ± 10.3 49.8 ± 8.8

**Work experience** (years) 19.3 ± 9.5 15.6 ± 10.1 20.4 ± 9.1 14.4 ± 6.2 19.6 ± 10.0 21.5 ± 8.7

**Specialised field**

Internal medicine 97 (29.2%) 28 (32.9%) 68 (28.9%) 1 (0.7%) 86 (95.6%)

Anaesthesiology 63 (19.0%) 26 (30.6%) 29 (12.3) 38 (76.0%)

Orthopedics 146 (44.0%) 19 (22.4%) 127 (54.0%) 4 (8.0%) 136 (97.8%) 3 (3.3%)

Other 26 (7.8%) 12 (5.1%) 11 (4.7%) 6 (12.0%) 2 (1.4%) 1 (1.1%)

**Employment centre**

University 37 (11.1%) 15 (17.6%) 20 (8.5%) 11 (22.0%) 7 (5.0%) 11 (12.2%)

District hospital 113 (34.0%) 32 (37.6%) 75 (31.9%) 27 (54.0%) 47 (33.8%) 18 (20.0%)

Private practice 160 (48.2%) 30 (35.3%) 129 (54.9%) 10 (20.0%) 77 (55.4%) 57 (63.3%)

Pain centre 2 (0.6%) 1 (1.1%) 1 (3.0%) 1 (1.1%)

Other 20 (6.0%) 7 (3.0%) 10 (4.3%) 2 (4.0%) 8 (5.8%) 3 (3.3%)

**Status of specialisation**

Resident 41 (12.3%) 16 (18.8%) 20 (8.5%) 11 (22.0%) 14 (10.1%) 2 (2.2%)

Consultant 281 (84.6%) 65 (76.5%) 210 (89.4%) 36 (72.0%) 125 (89.9%) 88 (97.8%)

**Treatment Ratio**

More than 4 patients per week 163 (49.1%) 34 (40.0%) 124 (52.8%) 26 (52.0%) 70 (50.4%) 44 (48.9%)

Up to 3 patients per week 87 (26.2%) 19 (22.4%) 62 (26.4%) 13 (26.0%) 36 (25.9%) 25 (27.8%)

Up to 3 patients per month 39 (11.7%) 18 (21.2%) 20 (8.5%) 7 (14.0%) 16 (11.5%) 9 (10.0%)

Up to 10 patients per year 36 (10.8%) 13 (15.3%) 23 (9.8%) 4 (8.0%) 13 (9.4%) 12 (13.3%)

Never 6 (1.8%) 1 (1.2%) 5 (2.1%) 4 (2.9%) 2 (2.2%)

**Referral Ratio**

More than 4 patients per week 26 (7.8%) 8 (9.4%) 18 (7.7%) 9 (6.5%) 15 (16.7%)

Up to 3 patients per week 26 (7.8%) 5 (5.9%) 20 (8.5%) 2 (4.0%) 9 (6.5%) 12 (13.3%)

Up to 3 patients per month 96 (28.9%) 24 (28.2%) 69 (29.4%) 6 (12.0%) 42 (30.2%) 36 (40.0%)

Up to 10 patients per year 89 (26.8%) 22 (25.9%) 61 (26.0%) 12 (24.0%) 37 (26.6%) 21 (23.3%)

Never 82 (24.7) 18 (21.2%) 61 (26.0%) 21 (42.0%) 42 (30.2%) 7 (7.8%)

Table S1 provides the demographic data of physicians dealing with myofascial pain: age, gender, field of specialisation, subspecialisation, employment centre and status of specialisation. We asked for the physician’s average number of treated myofascial pain patients (Treatment ratio) and the number of patients referred to specialised pain centres (Referral Ratio).Data are expressed as mean ± SD or as total count (n) and in percent (%).
