# Supplementary material for: Discrepancy between prevalence and perceived effectiveness of treatment methods in myofascial pain syndrome: Results of a cross-sectional, nationwide survey
Source: BMC Musculoskelet Disord. 2010 Feb 11;11:32. doi: 10.1186/1471-2474-11-32 (PMC2836281; doi:10.1186/1471-2474-11-32)
Supplement: Additional file 3 — Table S2 - Estimated prevalence (mean ± SD). Table S2 provides the physician estimated importance of myofascial pain in the general population on a six-point scale (with 1 being a "very common problem"). Additionally the physician estimated prevalence of active trigger points in the general population and their respective patients in percent (%) is given. Data are expressed as mean ± SD. [file 1471-2474-11-32-S3.DOC]

## Table S2 - Estimated prevalence (mean ± SD)

Subgroups .

All Female Male Pain therapists Rheumatologists Orthopaedists

n = 332 n = 85 n =235 n = 50 n = 90 n = 139

**Importance** (1-6) 2.5 ± 1.4 2.6 ± 1.4 2.5 ± 1.4 2.6 ± 1.3 2.5 ± 1.2 2.5 ± 1.5

**Prevalence of active trigger points (%)**

General population 46.1 ± 27.3 50.0 ± 23.3 44.7 ± 28.6 55.4 ± 22.2 36.3 ± 27.3 46.4 ± 27.5

Respective patients 52.6 ± 26.8 54.9 ± 24.9 51.8 ± 27.5 63.4 ± 21.7 47.6 ± 27.2 48.7 ± 26.8

Table S2 provides the physician estimated importance of myofascial pain in the general population on a six-point scale (with 1 being a “very common problem”). Additionally the physician estimated prevalence of active trigger points in the general population and their respective patients in percent (%) is given. Data are expressed as mean ± SD.
