# Supplementary material for: Discrepancy between prevalence and perceived effectiveness of treatment methods in myofascial pain syndrome: Results of a cross-sectional, nationwide survey
Source: BMC Musculoskelet Disord. 2010 Feb 11;11:32. doi: 10.1186/1471-2474-11-32 (PMC2836281; doi:10.1186/1471-2474-11-32)
Supplement: Additional file 4 — Table S3 - Prescription Rate of Treatment Options (in %). Table S3 indicates the physician estimated prescription rate of different therapeutic options in the treatment of myofascial pain. Data are expressed in percent (%). TENS: transcutaneous electrical stimulation. [file 1471-2474-11-32-S4.DOC]

## Table S3 - Prescription Rate of Treatment Options (in %)

Subgroups .

All Female Male Pain therapist Rheumatologists Orthopaedists

n = 332 n = 85 n = 235 n = 50 n = 90 n = 139

**Analgesics**

NSAIDs' and coxibs 91.6 91.8 91.1 92.0 89.3 94.2

Metamizol and paracetamol 87.0 85.9 87.2 82.0 90.3 89.2

Weak opioids 81.6 81.2 82.6 76.0 88.2 82.7

Antidepressants 72.3 76.5 69.8 72.0 92.5 59.0

Strong opioids 57.2 45.9 60.9 64.0 66.7 50.4

Anticonvulsants 52.7 51.8 52.8 52.0 67.7 43.2

Others 16.9 16.5 14.5 36.0 3.2 13.7

- Muscle relaxants 5.1 2.4 4.3 8.0 1.1 2.2

- Flupirtine 4.8 9.4 2.6 20.0 0.0 0.0

**Physical therapy**

Manual therapy 81.3 74.1 84.7 88.0 69.9 91.4

TENS 72.9 72.9 71.9 90.0 60.2 76.3

Acupuncture 60.2 55.3 60.9 72.0 38.7 70.5

Ultrasound 39.8 25.9 46.8 16.0 39.8 56.1

Percussion waves 31.9 14.1 39.6 16.0 22.6 48.9

Dry needling 28.9 22.4 30.2 46.0 15.1 30.9

Others 21.7 11.8 26.4 4.0 19.4 33.1

**Injections**

Injection of local anaesthetics 71.1 69.4 72.8 70.0 51.6 87.8

Spinal interventions 30.7 24.7 32.8 40.0 15.1 37.4

Injection of botulinum toxin 12.3 14.1 11.9 24.0 9.7 10.1

Others 3.3 2.4 3.4 4.0 2.9

Table S3 indicates the physician estimated prescription rate of different therapeutic options in the treatment of myofascial pain. Data are expressed in percent (%). TENS: transcutaneous electrical stimulation.
