# Supplementary material for: Discrepancy between prevalence and perceived effectiveness of treatment methods in myofascial pain syndrome: Results of a cross-sectional, nationwide survey
Source: BMC Musculoskelet Disord. 2010 Feb 11;11:32. doi: 10.1186/1471-2474-11-32 (PMC2836281; doi:10.1186/1471-2474-11-32)
Supplement: Additional file 5 — Table S4 - Ratings (1-6) of treatment options (mean ± SD). Table S4 indicates the physician estimated efficacy of different therapeutic options in the treatment of myofascial pain on a 6-fold scale (with 1 being "excellently effective" and 6 being "ineffective"). Data are expressed as mean ± SD. TENS: transcutaneous electrical stimulation. [file 1471-2474-11-32-S5.DOC]

## Table S4 - Ratings (1-6) of treatment options (mean ± SD)

Subgroups .

All Female Male Pain therapist Rheumatologists Orthopaedists

n = 332 n = 85 n = 235 n = 50 n = 90 n = 139

**Analgesics**

NSAIDs' and coxibs 2.7 ± 1.0 2.6 ± 1.0 2.8 ± 1.0 2.8 ± 1.1 3.1 ± 1.1 2.5 ± 1.0

Metamizol and paracetamol 3.1 ± 1.0 3.1 ± 0.9 3.1 ± 1.1 3.1 ± 0.9 3.2 ± 1.0 3.0 ± 1.1

Weak opioids 3.1 ± 1.1 2.9 ± 1.1 3.1 ± 1.1 3.7 ± 1.2 3.0 ± 1.1 2.8 ± 1.0

Antidepressants 2.6 ± 1.0 2.5 ± 1.1 2.7 ± 1.0 2.5 ± 1.0 2.6 ± 1.1 2.7 ± 1.1

Strong opioids 3.2 ± 1.5 3.2 ± 1.7 3.1 ± 1.4 3.9 ± 1.4 3.1 ± 1.5 2.7 ± 1.3

Anticonvulsants 3.2 ± 1.2 3.0 ± 1.3 3.2 ± 1.2 3.7 ± 1.2 3.0 ± 1.1 2.9 ± 1.3

Others 2.3 ± 1.1 2.0 ± 0.8 2.5 ± 1.2 2.0 ± 0.7 3.3 ± 1.5 2.8 ± 1.1

- Muscle relaxants 2.1 ± 0.5 2.0 ± 0.0 2.1 ± 0.6 1.8 ± 0.5 3.0 2.0 ± 0.0

- Flupirtine 1.6 ± 0.7 1.6 ± 0.7 1.3 ± 0.5 1.7 ± 0.7

**Physical therapy**

Manual therapy 2.3 ± 0.9 2.0 ± 0.8 2.3 ± 0.9 2.2 ± 0.8 2.6 ± 1.0 2.2 ± 0.8

TENS 2.6 ± 0.9 2.5 ± 1.0 2.6 ± 0.9 2.4 ± 0.6 3.0 ± 0.9 2.5 ± 0.9

Acupuncture 2.4 ± 1.0 2.4 ± 0.9 2.5 ± 1.0 2.4 ± 1.1 3.0 ± 1.1 2.3 ± 0.8

Ultrasound 3.0 ± 1.2 2.8 ± 1.1 3.1 ± 1.2 3.6 ± 1.1 3.2 ± 1.2 2.9 ± 1.1

Percussion waves 2.8 ± 1.2 3.0 ± 1.1 2.8 ± 1.2 3.3 ± 1.5 3.3 ± 1.4 2.6 ± 1.1

Dry needling 2.4 ± 1.1 2.5 ± 1.1 2.4 ± 1.1 2.0 ± 1.2 2.6 ± 0.8 2.6 ± 1.2

Others 2.0 ± 0.9 2.0 ± 1.5 2.0 ± 0.8 3.5 ± 3.5 2.3 ± 0.8 1.9 ± 0.8

**Injections**

Injection of local anaesthetics 2.3 ± 1.0 2.2 ± 1.1 2.4 ± 1.0 2.3 ± 1.0 2.8 ± 1.1 2.2 ± 0.9

Spinal interventions 2.9 ± 1.8 3.7 ± 2.0 2.8 ± 1.7 3.5 ± 1.8 3.3 ± 1.4 2.6 ± 1.6

Injection of botulinum toxin 2.8 ± 1.4 3.0 ± 2.1 2.8 ± 1.1 2.9 ± 1.4 3.4 ± 1.9 2.1 ± 0.8

Others 1.9 ± 0.5 2.0 ± 0.0 1.9 ± 0.6 2.5 ± 0.7 1.8 ± 0.5

Table S4 indicates the physician estimated efficacy of different therapeutic options in the treatment of myofascial pain on a 6-fold scale (with 1 being “excellently effective” and 6 being “ineffective”). Data are expressed as mean ± SD. TENS: transcutaneous electrical stimulation.
